# Problems and issues in implementing innovative curriculum in the developing countries: the Pakistani experience

**DOI:** 10.1186/1472-6920-12-31

**Published:** 2012-07-12

**Authors:** Syeda Kauser Ali, Lubna A Baig

**Affiliations:** 1 Department for Educational Development, Aga Khan University, Karachi, Pakistan; 2 Medical Education, King Saud Bin Abdulaziz University of Health Sciences, Riyadh and Medical Education and Research Unit, University of Calgary, Calgary, Canada

**Keywords:** Community-oriented medical education, Curriculum development, Educational innovation, Change management, Evaluation of curriculum, Educational leadership

## Abstract

****Background**:**

The Government of Pakistan identified 4 medical Colleges for introduction of COME, one from each province. Curriculum was prepared by the faculty of these colleges and launched in 2001 and despite concerted efforts could not be implemented. The purpose of this research was to identify the reasons for delay in implementation of the COME curriculum and to assess the understanding of the stakeholders about COME.

****Methods**:**

Mixed methods study design was used for data collection. In-depth interviews, mail-in survey questionnaire, and focus group discussions were held with the representatives of federal and provincial governments, Principals of medical colleges, faculty and students of the designated colleges. Rigor was ensured through independent coding and triangulation of data.

****Results**:**

The reasons for delay in implementation differed amongst the policy makers and faculty and included thematic issues at the institutional, programmatic and curricular level. Majority (92% of the faculty) felt that COME curriculum couldn’t be implemented without adequate infrastructure. The administrators were willing to provide financial assistance, political support and better coordination and felt that COME could improve the overall health system of the country whereas the faculty did not agree to it.

****Conclusion**:**

The paper discusses the reasons of delay based on findings and identifies the strategies for curriculum change in established institutions. The key issues identified in our study included frequent transfer of faculty of the designated colleges and perceived lack of:

· Continuation at the policy making level

· Communication between the stakeholders

· Effective leadership

## **Background**

Curricular review, revisions and modifications have been routine practice in medical institutions of the developed countries. Recently developing countries are also experimenting with different curricular models. However, initiating, implementing and sustaining change has not been easy [1,2]. Successful educational improvements require establishing a clear educational vision and a shared institutional mission. According to Bland (2000) [3] “successful curricular change occurs only through the dedicated efforts of effective change agents”.

The authors report on the process of change and the elements necessary for effective change from the standpoint of a governmental decree for change in multiple (four) medical schools. In addition to the perspective about the country wide and government mandated change process, there is a unique political situation included in the change process; specifically the instability in the government of Pakistan at the time the changes were attempted. This is an aspect of change that is not usually reported in the literature from the developed world.

Of the 35 features of successful curricular change identified by Bland et al. we used organization's mission and goals, internal networking, resource allocation, relationship with the external environment, organizational structure, need for change, scope and complexity of the innovation, cooperative climate, participation by the organization's members, communication, human resource development, evaluation, and leadership to identify the factors that were hindering the smooth implementation of the curricular change [3].

We also describe a process that did not work as planned and the reasons for that. There is too little published about what does not work and we hope that our study will provide useful insight into why a planned activity did not work and the lessons that can be learned from the unanticipated outcomes.

A change was instituted by the government of Pakistan in 1992 taking lead from the Edinburgh Declaration asking the faculty and administrators of medical institutions to review the existing medical curriculum and develop a revised/new curriculum for use by all the medical institutions of the country. The World Health Organization (WHO) was contacted for assistance in this regard and a series of deliberations and discussions were held with educational leaders who had successfully introduced curricular innovations in the countries with similar health care problems. In the light of these deliberations and educational philosophies [4], best evidence medical education practices (BEME), and the health problems and health delivery structure of the country, a Community-Oriented Medical Education (COME) curriculum with Problem-based learning (PBL) as the instructional methodology was finally selected. The COME project (as it was called) was initiated as a pilot in 1994 in collaboration with the WHO by the Government of Pakistan.

Four medical Colleges, one from each province were included in the program. They were Dow Medical College (Sindh), King Edward Medical College (Punjab), Bolan Medical College (Balochistan) and Ayub Medical College (Khyber Pakhtoon Khwah). The faculty of medical colleges and the other stake holders (medical students, representatives of ministry of health and service providers from potential catchment areas who were to become partners in student learning) were involved in revising the traditional curriculum with incorporation of COME in undergraduate teaching [5].Medical students were also involved in this process and participated in the introductory curriculum development workshops which introduced the participants to the educational principles and PBL process. Members from the ministry of health were invited on the first and last day of the workshops to show their commitment, to know the views of the faculty, review the changes recommended in the curriculum as a result of the workshop process and discuss their own role in the implementation process. Large full day meetings were held in the selected community sites to initiate the process of developing partnership with these communities. These meetings were attended by service providers, community leaders, elected councilors, school teachers and representative from community based organizations (CBOs). These meetings were conducted by the national and provincial COME coordinators, national and international consultants in consultation with the Provincial Minister of Health and Principal^1^ of the COME College.

The conceptual framework for the curriculum was taken from the spiral curriculum at the medical school of Dundee in United Kingdom [6,7]. A consensus was reached by the faculty of COME colleges in the initial workshops that the medical graduate should be a safe, skillful, and humane medical practitioner [5]. The curriculum was distributed over five years in three phases with horizontal and longitudinal blocks as follows:

**Phase 1** consisted of year: 1 and 2 of medical college and included Normal structure and function, abnormal structure and function with clinical relevance.

**Phase 2** consisted of year 3 and included abnormal structure, function and patient management with clinical rotations in major disciplines

**Phase 3** consisted of rotations and training in the hospital in all required disciplines as per the core competencies: defined by the Pakistan Medical and Dental Council (clerkship)

The two longitudinal blocks were:

**Community Experience** was over the four year period (1st to 4th year) with the final exam in the fourth year

**Clinical skills** started in the 1st year and continued till the final year with varying levels of competency training.

A coordinated and integrated approach was adopted for developing the curriculum [2,5,8] learning from the experience of the Interdisciplinary Generalist Curriculum (IGC) Project of North America [9,10].A national coordinator (similar to the IGC project officer), international and a national consultant, and four provincial coordinators were deputed to oversee the academic and administrative aspects of the program, and facilitate transition from traditional to innovative curriculum [11].The provincial coordinators were selected from the medical institutions within the program [11,12]. The curriculum was developed during regular meetings of the faculty from these four colleges and launched in 2001. Three out of these four colleges piloted one to two first year blocks. However despite intensive efforts by the consultants, coordinators, and the faculty, the COME curriculum could not be implemented till 2004. Recently some aspects of the curriculum i.e. thematic curriculum using case-based learning (CBL) have been introduced in at least one medical college.

A study was initiated by WHO and Ministry of Health (MOH) in 2004 to look into the factors, which hampered the implementation of the COME curriculum in the selected colleges. The objectives of the study were to identify the reasons for non implementation of the COME Project and to assess the understanding of the stakeholders about COME.

## **Methods**

A mixed method approach was used with both quantitative and qualitative study designs. Data collection was done by detailed interviews, mail-in survey questionnaire and discussions with groups of stakeholders.

Detailed interviews were conducted using a semi structured interview guide by one of the authors to maintain continuity. The interviews were requested from 20 key informants including officials of the federal and provincial health department, administrative heads/Principals of medical colleges, coordinators and consultants of the COME program to identify their knowledge of the COME project, their required roles for implementation and views about the factors that hindered the implementation process. It was important to conduct interviews with this group since they were not willing to complete the questionnaire.

Survey questionnaire consisting of two distinct segments was mailed to all faculty members of the four medical colleges selected for piloting the COME project. One segment of questionnaire enquired about the knowledge, practices and attitude of the faculty member with respect to medical education itself and the academic structure of their medical college. This section consisted of 12 statements where the respondents had to select as many options as were appropriate. The second segment consisted of 20 statements regarding the implementation process and faculty (the respondents) had to indicate their level of agreement/disagreement on a five-point Likert scale. The faculty was asked to respond anonymously and return the completed questionnaires in a pre paid envelope back to the evaluator (first author).

Focus group discussions (FGDs) were held by the co evaluator (second author) with first year medical students in all four medical colleges. Each focus group consisted of 6–8 students selected purposively. The students participating in the faculty development workshops and identified by the faculty as opinion leaders from the Medical College were asked to participate in the focus groups. No one was forced to come and the participation was voluntary. The objective was to find out their knowledge of and perspectives regarding COME, PBL, student involvement in the change process, their views on student assessment and adequacy of available resources for the new curriculum. The FGDs were also held with volunteering faculty in all four colleges on the same areas. Each focus group consisted of 10–12 faculty members with an aim to obtain their perspective on implementation and their apprehensions regarding the COME curriculum.

None of the medical colleges under study had an ethics review board at that time and hence the study was sent to the WHO –EMRO and the Federal Health Ministry of Pakistan for approval. The study was started only after approval from WHO –EMRO and the Federal Ministry of Health Pakistan.

### **Analysis**

The interviews and focus group were recorded verbatim by the concerned interviewer/evaluators. The records were duplicated with one copy given to each evaluator. The evaluators individually coded the responses and discussed the codes and categorization. Both evaluators discussed the codes and the three themes emerging from them. The Evaluators then reviewed the interviews again and a point of saturation was ensured if the same themes and sub-themes were identified in the second coding. Bland’s groups of issues related to planning and implementation were addressed in all the three themes [3,13]. The three themes with their respective sub-themes included:

· Institutional issues in implementation

· Infrastructure

· Faculty development

· Faculty apprehensions

· Programmatic issues in implementation

· Organization and coordination

· Financial support

· Political commitment

· Effect on health system of the country

· Need and usefulness of COME curriculum

· Faculty readiness and knowledge of the program

· Curricular issues in implementation

· Community based learning

· Problem-based learning

· Participation of students in curriculum planning and development

· Student assessment

· Resources

The codes and themes were then discussed together and any differences were sorted out by going back to the primary documents and mutually agreeing on the major theme. Faculty survey questionnaire and the focus group with students were used for triangulation of the themes and sub-themes. This was done first independently by the two evaluators and then together a consensus was reached to ensure rigor of study and reliability of data.

## **Results**

The results section is organized under four major headings, the first section has details on the sample and interviewees, second section is on the first theme, the institutional issues, the third section is on programmatic issues and the final section deals with the curricular issues. The percentages wherever reported, in the text and tables, are from the survey questionnaire.

1. Interviewees information Out of the 20 key informants contacted for detailed interview 16 were available for interview. Saturation was reached by the 12th interview however we continued with the remaining four. (refer to Table 1 for information regarding the key informants) Response rate of the survey questionnaires ranged from 70% to 85% from the four medical colleges (refer to Table 2).

2. Institutional issues There were differences in the opinion of the administrators/managers of the program and the actual implementers namely the faculty. Most (65%) of the faculty felt that teachers had not been adequately trained in conducting PBL sessions, whereas the administrators insisted that teachers were adequately trained. There was also a perception by faculty (30%) that the program was not going to address national needs but was being imposed by the WHO. Regarding the coordination of the COME program, 51% of the faculty felt that it lacked appropriate management at the institutional level, while 47% felt that it was not well managed at the provincial level. Overall 78% of the faculty was in favor of COME and PBL but a large majority of the faculty (92%) felt that COME cannot be implemented without an adequate infrastructure and were of the view that the implementation should be delayed until all the infrastructure requirements are completed. The administrators at the MOH and WHO felt that they have provided adequate support and are willing to do so. Whereas the faculty felt that the major cause of delay is due to inadequate support, fear of failure, lack of reward for increase in workload, and other administrative matters (refer to Table 3).

3. Programmatic issues The programmatic issues identified by the faculty and the administrators of the program differed widely with the responsibility of delay being levied on to the other group. The administrators felt that they were providing the needed financial assistance, political support and coordination whereas the faculty perception was contrary to it (refer to Table 4). The administrators were of the view that implementation of COME will have a positive effect on the health services and systems whereas the faculty felt that this was not possible by a curricular change only. Overall 64% of the faculty was prepared to take the students to the community and 62% were ready to give 10% to 30% of their working hours in the community. More than 60% of the faculty agreed that they understand the role of community-based education in the Pakistani context, while 42% said that they were not well informed about the progress of the COME program (refer to Table 4).

4. Curricular issues The curricular issues were addressed with the faculty and the students. The new curriculum was supported and favored by 29% of faculty while 41% were unsure about its usefulness. The students liked PBL process but were unsure about the value of new curriculum and felt that they were being experimented upon and their future is being put at stake. Of the faculty who responded to the questionnaire 65% commented that they were happy with the improvement in medical education. The assessment system at the university was identified as a major obstacle in implementing the curriculum since the assessment system did not match the innovative teaching methodology. (refer to Table 5).

**Table 1:** Details of key informants approached for interview

***Designation of informants***	***Contacted (n = 20)***	***Interviewed (n = 16)***
***DG health***	***1***	***Deputy DG health***
***Provincial health ministers***	***4***	***2***
***Provincial health secretaries***	***4***	***3***
***Principals of medical colleges***	***4***	3 + 1*
**COME consultant (international)**	***1***	***1***
***National COME coordinator***	***1***	***1***
***COME coordinators (institutional)***	***4***	3 + 1*
***Total***	***20***	***16***

* The principal and coordinator of an institution insisted on having combined interview.

**Table 2:** Percentage of respondents to the survey questionnaire

Medical college	Total Faculty	Number responded	%
Ayub Medical College	142	115	81
Bolan Medical College	139	97	70
Dow Medical College	235	190	81
King Edward Medical College	144	122	85
Total	660	524 Response rate 78%	

**Table 3:** Institutional Issues in Implementation identified on the basis of questionnaire (completed by the faculty) and key informant interviews (with the administrators)

Faculty Perceptions n = 514	Administrators Perception n = 16
Infrastructure	
· Need adequate infrastructure like library, skills lab, rooms for small group discussion with teaching aids (92% felt it is a must).	· The concerned Ministry was willing to provide financial support for infrastructure however they needed proper documentation (5/16).
· WHO has not provided adequate support for the equipment (38%).	· WHO has not provided adequate support for the equipment (4/16).
Faculty Development	
· Adequate number of teachers not trained in conducting PBL sessions (65%).	· Teachers adequately trained, institutional changes take time (10/16).
· WHO has not provided adequate support for training (40%).	· WHO only provides for technical assistance, however training support was adequate (3/16).
· Adequate number of teachers not aware of PBL principles and concepts (65%).	· Due to slow progression of COME the faculty enthusiasm is dying off (6/16).
· Transfer of trained faculty to other institutions. (65%)	· Transfer of trained faculty to other institutions (4/16).
· Lack of fellowships by WHO (54%).	
Faculty Apprehensions	
· Increase work load for the faculty (70%).	· Time consuming hence incentives need to be added (4/4 principals of COME colleges mentioned it).
· Fear of failure (reverting back to the traditional curriculum) (55%).	· No fear of failure (3/4 principals).
· The program has been imported from the west and hence not suitable for our educational system (30%).	· The program has been initiated after the government signed the Edinburgh Declaration and acquired WHO support (1/16).
· The program has not been initiated in consultation with concerned faculty; it has been forced on us because of WHO pressures (20%).	

**Table 4:** Programmatic Issues in Implementation

Faculty Perceptions	Administrators Perception
Organization and Coordination	
· Lack of coordination between the ministry, institutions, health departments and WHO (95%).	· Lack of coordination between the ministry, institutions, health departments and WHO (4/16 all principals).
· Faculty not informed of the progress on COME (71%).	· Not sure of the time lines on implementation (9/16).
· Faculty not informed of proposed time of implementation (68%).	· Why did the faculty not think about the evaluation issue earlier on (4/16 – administrators at provincial level).
· Transfer of trained faculty caused delays in implementation (55%).	
· The assessment system by the university is not congruent with the PBL and COME curricula (20%).	
Financial support	
Lack of financial support for photocopying, books, petrol for students’ community visit, secretarial support and faculty incentives (99%).	· The concerned Ministry was willing to provide financial support however they needed official documentation from the principal (5/16 administrators at provincial and federal level).
Political Commitment	
· The principals were not in favour of COME (57%).	· They felt that the principals were not complying (5/16 administrators at provincial and federal level).
· Lack of political commitment (30%).	
· Frequent change of administrator at all levels (35%).	· No lack of political commitment, government is fully supportive (5/16 administrators at provincial and federal level).
· Lack of ownership by the provincial government (35%).	· Frequent change of administrator at all levels (10/16).
	Lack of directive from the federal ministry (4/16 all principals).
Effect on Health System of the Country	
· No effect on health system of the country (54%).	· The health system of the country will improve with implementation (9/16).
· The senior faculty does not have time to go the field site and are not trained to go in the field (47%).	· The senior faculty will come in contact with the service providers at the peripheral level with a hope to improve their competencies (3/16).
· The community comes to the tertiary care teaching hospitals; hence the students are adequately trained (45%).	· The cost of in-training of medical doctors after posting to Basic Health Units will be decreased (3/16).
	· Tertiary care teaching institutions will be linked with the community health services (3/16).
Need and Usefulness of COME	
· We do not need to send the students to the community because the community comes to our hospitals (74%).	· Unless the infrastructure in Community health services is organized to receive students for medical training, it will be difficult to implement COME in Pakistan (3/16).
· Infrastructure of the community is not developed and the staff is not trained in the peripheral centers (92%).	· Presence of students will have beneficial effect on the practices of the health providers at the primary care level (3/16).
Faculty readiness and knowledge of the program	
· Lack of acceptance by the faculty at large for the change (12%).	· Not sure of the abbreviation and concept of COME (3/16 administrators).
· Some faculty members did not know what the abbreviation COME stood for (8%).	· COME means taking students to the community by the department of community Medicine (6/16 administrators).
· We are ready to take the students to the community for learning (64%).	

**Table 5:** Curricular Issues in Implementation identified on Focus Group Discussion

Students Perceptions	Faculty Perceptions
Community-based learning	
·We do not understand what is expected of us in the community.	·Students will greatly benefit from the community - based activities (66%).Since the students will learn in the setting where most of them will practice after graduation they will be better equipped to deal with health problems at the community level (60%).
Problem-based learning	
·PBL sessions are enjoyable.	·Students are going to enjoy the PBL method (70%).
·The learning is relevant to our future practices.	
Participation of students in curriculum planning and development	
·It is our future and we have not been consulted.	
·We are being treated as experimental subjects.	
·Lack of continuity in institutional policy.	
Students Assessment	
(As there were more than one medical college under the same university, hence the university assessment systems continues to be on the traditional discipline-based pattern)	
·“We will be disadvantaged as the students coming from traditional colleges will do better in the exams”.	·Lack of agreement with University regarding evaluation system (54%).
·“The value of the degree may be jeopardized as the teaching methodology is different from other medical institutions of the country”.	
Resources	
·Lack of material for learning through PBL.	·Lack of support staff and stationary (70%).
·Absence of books that follow the problem-oriented approach	·Lack of books in the library (79%).
·Teachers not trained.	
·Inadequate infrastructure of the college.	

## **Discussion**

Studies of successful curricular changes have emphasized that such modifications should focus on all aspects of education from the curriculum to: assessment, teacher preparation, school calendar, content structure, educational context, organizational structure and institutional culture [14-17]. Each of these components is interconnected and the institutional commitment to the innovation is essential in order to see the process through. Communication, coordination and cooperation of the Institutional and departmental heads is essential for successful change as is willingness to make corrections during the process [11,18].

Faculty development helps to provide teachers with the capacity to implement and support the efforts for educational reform [19]. In this case faculty development was addressed through teacher-training workshops, however the results showed that a large number of faculty members felt that they were not involved in the process and were not adequately prepared. This apprehension of the faculty seems speculative, as it has been reported that once the curriculum revision progresses the faculty development ensues as an intended or unintended change [20-22]. The possible reasons for this perception could be lack of communication between the project managers and the faculty as well as frequent transfers of the faculty to and from other medical institutions that were not included in the pilot. This added on to the apprehension of the already perplexed faculty still feeling ill-equipped and ready to take on the challenges of curriculum change. This faculty backlash was not envisioned by the administrators and could have been managed better if noted earlier [18].Having open communication channels have been identified as one of the most important elements in change management which seems to be compromised in planning and implementation of this project [23].

During this time the political environment of country was volatile and leadership with the political parties changed numerous times between the years 1994 to 2000. This led to frequent changeover of the ministers, secretaries and other officials of the government which also led to changes in the project leadership. The national coordinator for the COME program changed 3–4 times that also made smooth and speedy implementation next to impossible. The same happened in case of the institutional heads and many changed over this period of time. The commitment of institutional head has been considered as an essential factor in successful curricular changes internationally [11,24,25]. As a result of these frequent changes of personnel the vision of the COME project did not cascade to the actual implementers and demonstrates a lack of continuity of leadership to institute the change [23,26].

Historically established institutions of long standing have revised their curricula with participation of all stakeholders. The institutions working through a process of faculty buy-in and acceptance [27] introduced the curriculum using phased-in approach or introduced a parallel track during transition from traditional to innovative curricula [8,19,26,28]. While studying this program it occurred on many occasions that the planning was not clear or was not clearly communicated to the implementers and practitioners of the COME program. The planners were trying to bring about major changes in the curriculum in established institutions with traditional curriculum. Studies of successful curricular change have shown that good communication and proper planning is a key to success [12].

Any educational change entails cost and although the analysis of the data shows that the commitment for continuing the program was present at the governmental and institutional level, the institutional heads had reservations about its sustainability [10,24]. Reports of successful implementation of the IGC project, USA demonstrated that small but continuous monetary support helped in the smooth implementation of the project leading to desired results. However, despite heavy input of monetary support by the MOH and WHO; lack of adequate resources and funding was identified as a major issue [3,13,24]. Another major difference in the two projects was that the external evaluators/inspectors were not visiting the project site in the COME project for monitoring progress as they did in the IGC project [24,25]. Lack of adequate monitoring, prompt and appropriate tackling of the issues as they arose were also possibly contributing to the delay in implementation of the project.

Assessment needs to change along with instruction to ensure that what is being assessed is what is taught [14-16,29].Three medical colleges out of four were under the administrative control of universities that had other affiliated medical colleges, which were following the traditional curriculum. The university assessment continued to be on the traditional discipline-based design not in conformity with the innovative curricula proposed by COME.

One of the main limitations of the study was small number of students and faculty that were involved in FGDs. Another one was that the questionnaire and interview guide were not pre-tested

## **Conclusions**

Our study reinforces the idea that the elements necessary to accomplish change are universal. Although there was the added constraint of political instability in the country, all other elements necessary for the change process support the findings of other studies. The study identifies the problems and pitfalls that lie in attempting to incorporate a community component to the medical education program and we feel that the experience documented in the article would be useful for others, regardless of the country in which they are attempting to incorporate the change. One of the unique finding in our study is frequent transfers of faculty from one institution to another with a resulting faculty turn over and a need for continued faculty training activities.

In the light of this study we have learnt that any curriculum change management plan should ensure:

· Continuity of polices and commitment at governmental level

· Shared vision of faculty and administrators

· Continuity of committed faculty

· Gradual phase-in of the curricular change ensuring that all modifications are appropriate inclusive of the student assessment system

· Provision for continued financial and technical support

· Monitoring of the process of implementation at the programmatic and institutional level from the initiation of the project

· Participation and involvement of all stakeholders at all levels from the beginning of the planning process

## Endnotes

^1^The administrative head of the medical college in Pakistan is the Principal

## Competing interests

There is no known conflict of interest and the study was conducted at the behest of Ministry of Health Government of Pakistan and World Health Organization.

## Authors’ Contribution

Dr. Ali SK conceptualized the study, designed the questionnaire, did the interviews, transcribed and analyzed the data and wrote the first draft. Dr. Baig LA helped in the design of the study, conducted the focus groups, analyzed the data and worked equally on the paper. Both author read and approved the final manuscript.

## Pre-publication history

The pre-publication history for this paper can be accessed here:

http://www.biomedcentral.com/1472-6920/12/31/prepub
